# Editorial: Combination of photodynamic therapy and immunotherapy to overcome cancer resistance

**DOI:** 10.3389/fimmu.2026.1833324

**Published:** 2026-04-01

**Authors:** Mojtaba Hoseini-Ghahfarokhi, Kolawole A. Olofinsan, Paromita Sarbadhikary, Glory Kah, Precious Earldom Mulaudzi, Kave Moloudi

**Affiliations:** 1 MD Anderson Cancer Center, University of Texas, Houston, TX, United States; 2 Department of Pharmacology, Faculty of Health Sciences, University of the Free State, Bloemfontein, South Africa; 3 Laser Research Centre, Faculty of Health Sciences, University of Johannesburg, Johannesburg, South Africa; 4 Department of Medical Microbiology, School of Pathology, Faculty of Health Sciences, University of the Free State, Bloemfontein, South Africa; 5 University of Caen Normandie, UNICAEN, UNIROUEN, ABTE UR4651, Cancer Center François Baclesse, Caen, France

**Keywords:** cancer resistance, immunotherapy, nanomedicine, photodynamic therapy, photoimmunotherapy, tumor microenvironment

## 1 Introduction

Cancer resistance remains a major challenge in oncology, limiting the effectiveness of many conventional therapeutic strategies such as chemotherapy and radiotherapy. In recent years, the integration of photodynamic therapy (PDT) with immunotherapy has emerged as a promising approach to overcome tumor resistance and enhance anticancer efficacy. PDT is a minimally invasive treatment that involves the activation of a photosensitizer by light in the presence of oxygen, leading to the generation of reactive oxygen species (ROS) that induce tumor cell death, vascular damage, and local inflammatory responses. Importantly, PDT can also trigger immunogenic cell death, which promotes the release of tumor-associated antigens and danger-associated molecular patterns, thereby stimulating the host immune system. Immunotherapy, including immune checkpoint inhibitors, cancer vaccines, and adoptive cell therapies, aims to activate and strengthen the immune system to recognize and eliminate malignant cells. However, many tumors develop mechanisms to evade immune surveillance, resulting in limited therapeutic responses. The combination of PDT and immunotherapy offers a synergistic strategy in which PDT enhances tumor antigen presentation and immune activation, while immunotherapy amplifies and sustains systemic antitumor immunity. Moreover, recent advances in nanotechnology and targeted drug delivery systems have further improved the therapeutic potential of this combination by enabling precise delivery of photosensitizers and immunomodulatory agents to tumor tissues. Therefore, integrating photodynamic therapy with immunotherapy represents a promising therapeutic paradigm for overcoming cancer resistance and achieving durable antitumor responses in precision oncology.

## 2 Editorial overview

Cancer resistance to conventional therapies remains one of the major challenges in oncology, often resulting from tumor heterogeneity, immune evasion, and adaptive survival mechanisms (1). In recent years, innovative therapeutic approaches such as photodynamic therapy (PDT) and cancer immunotherapy have emerged as promising alternatives for improving treatment outcomes. PDT involves the activation of a photosensitizer by light at a specific wavelength, leading to the generation of reactive oxygen species (ROS) that induce tumor cell death and vascular damage. Importantly, PDT can also stimulate local inflammation and trigger immunogenic cell death, which enhances antitumor immune responses (2, 3).

Cancer immunotherapy, including immune checkpoint inhibitors, monoclonal antibodies, and therapeutic vaccines, works by harnessing the patient’s immune system to recognize and eliminate tumor cells. However, many tumors develop mechanisms to evade immune surveillance, such as the creation of an immunosuppressive tumor microenvironment or the expression of inhibitory immune checkpoints (4, 5).

The combination of PDT with immunotherapy that often referred to as photoimmunotherapy—has attracted increasing attention as a synergistic therapeutic strategy. PDT can enhance antigen presentation, stimulate dendritic cells, and activate cytotoxic T lymphocytes, thereby converting tumors into *in situ* vaccines that amplify systemic antitumor immunity. When combined with immunotherapeutic agents such as checkpoint inhibitors or immune adjuvants, this approach can overcome tumor immune resistance, inhibit metastasis, and improve long-term treatment efficacy (6).

Recent advances in nanotechnology, biomaterials, and targeted drug delivery systems have further expanded the potential of PDT-based immunotherapy. Nanocarriers, antibody-photosensitizer conjugates, and smart biomaterials enable precise delivery of therapeutic agents to tumors while simultaneously activating immune responses. These integrated strategies have shown promising results in preclinical and translational studies, suggesting a powerful pathway toward personalized cancer therapy (7).

However, the primary remaining challenges in cancer drug resistance center on acquired resistance mechanisms, tumor heterogeneity, and microenvironmental factors that enable cancer cells to evade therapy. Multiple recent studies identify consistent obstacles and emphasize that genetic mutations, activation of alternative signaling pathways, drug efflux pumps, DNA repair mechanisms, and bypass signaling pathways drive resistance (8, 9). Kumar et al., specifically highlights tumor heterogeneity, cellular plasticity, cancer stem cells, and microenvironmental influences as major challenges (10). Moreover, Zugazagoitia et al., identified tumor heterogeneity and acquired resistance as probably the main limitation for the success of precision oncology (11).

## 3 Mechanistic synergy

### 3.1 Immunogenic cell death and danger signal release

PDT-immunotherapy combinations overcame cancer resistance through multiple synergistic mechanisms centered on immunogenic cell death (ICD) induction. PDT triggered robust antitumor immune responses (12) by releasing tumor-associated antigens and danger signals (13). Li et al. demonstrated that ER-targeting PDT induced robust ER stress and calreticulin (CRT) exposure on cell surfaces (14), which acted as an “eat me” signal to stimulate dendritic cell antigen-presenting function (14). Duan et al. showed that PDT using ZnP@pyro nanoparticles induced danger signals like CRT exposure and production of pro-inflammatory cytokines including TNF-α, IL-6, and IFN-γ (14), enhancing tumor immunogenicity and promoting immune cell infiltration (14).

### 3.2 Tumor microenvironment modification

PDT fundamentally altered the tumor microenvironment to enhance immunotherapy responsiveness. The therapy induced tumor antigen cross-presentation and T-cell activation (13), releasing antigens for cross-presentation to immune cells (13). Li et al. demonstrated that ER-targeting strategies induced specific ER stress and promoted immune activation (14), with enhanced anti-tumor efficacy compared to non-targeting systems providing evidence of synergistic rather than merely additive effects (14). Dąbrowski et al. showed that PDT’s modulation of inflammatory response affected the tumor microenvironment and determined susceptibility to systemic PD-1/PD-L1 inhibition (14).

#### 3.2.1 CD8+ T-cell responses and adaptive immunity

CD8+ T-cell activation emerged as a critical mechanism underlying combination therapy efficacy. Kleinovink et al., demonstrated that therapeutic effects were abrogated upon systemic depletion of CD8+ T cells, indicating PDT-induced tumor antigen cross-presentation and T-cell activation were essential (13). CD8+ T cells proved crucial for tumor eradication and delay in outgrowth of distant tumors (13). Kleinovink et al., showed that PDT induced a significant CD8+ T-cell response against tumors, which was further increased when combined with SLP vaccination and essential for therapeutic effect (13). Li et al. reported that ER-targeting PDT promoted CD8+ T cell proliferation and cytotoxic cytokine secretion (14). Duan et al. found enhanced CD8+ T-cell infiltration and activity in distant tumors (14).

#### 3.2.2 Checkpoint blockade enhancement

PDT created conditions that sensitized tumors to checkpoint inhibition. Gao et al. showed that inactivating IDO-1 overcame PDT-triggered adaptive immune resistance (12), with the combination provoking augmented antitumor immunity compared to PDT alone (12). This synergistic effect extended beyond merely additive benefits. Duan et al. demonstrated that immunogenic ZnP@pyro PDT treatment sensitized tumors to checkpoint inhibition mediated by PD-L1 antibody, not only eradicating primary tumors but also significantly preventing metastasis (14). The therapy increased other immune cell involvement, with enhanced NK cell and B cell infiltration into tumors (14).

#### 3.2.3 Mechanistic pathways of immune activation

The combination strategies activated multiple immune pathways. Hua et al. outlined how PDT induced ICD with release of tumor-associated antigens and damage-associated molecular patterns (DAMPs) (13). These danger signals activated immune pathways including cytokine release and involvement of antigen-presenting cells like dendritic cells (13), which processed antigens and presented them to T cells, leading to proliferation and differentiation of cytotoxic T lymphocytes (13). The synergistic effects of combining PDT with immune checkpoint blockade were evident in improved inhibition of tumor metastasis and recurrence (13), with biomarkers of immune activation including DAMP release and CTL involvement indicating robust immune responses (13).

The convergence of evidence across diverse PDT photosensitizers (pyrolipid (14), bacteriochlorin (14), Bremachlorin (13), ICG (14)) and multiple immunotherapy approaches (checkpoint blockade, vaccination, IDO inhibition) suggests the underlying immunogenic mechanisms of PDT are robust and compatible with various immune enhancement strategies. This breadth of successful combinations indicates the approach has potential applicability across multiple cancer types, though translation to clinical contexts will require validation in human subjects.
